# The Roles of GABA in Ischemia-Reperfusion Injury in the Central Nervous System and Peripheral Organs

**DOI:** 10.1155/2019/4028394

**Published:** 2019-11-11

**Authors:** Chaoran Chen, Xiang Zhou, Jialiang He, Zhenxing Xie, Shufang Xia, Guangli Lu

**Affiliations:** ^1^Institute of Nursing and Health, College of Nursing and Health, Henan University, Henan, Kaifeng, China; ^2^School of Food and Bioengineering, Henan University of Science and Technology, Luoyang, Henan, China; ^3^School of Basic Medicine, Henan University, Jinming Avenue, 475004 Henan, Kaifeng, China; ^4^Wuxi School of Medicine, Jiangnan University, Wuxi, China; ^5^Institute of Business, School of Business, Henan University, Henan, Kaifeng, China

## Abstract

Ischemia-reperfusion (I/R) injury is a common pathological process, which may lead to dysfunctions and failures of multiple organs. A flawless medical way of endogenous therapeutic target can illuminate accurate clinical applications. *γ*-Aminobutyric acid (GABA) has been known as a marker in I/R injury of the central nervous system (mainly in the brain) for a long time, and it may play a vital role in the occurrence of I/R injury. It has been observed that throughout cerebral I/R, levels, syntheses, releases, metabolisms, receptors, and transmissions of GABA undergo complex pathological variations. Scientists have investigated the GABAergic enhancers for attenuating cerebral I/R injury; however, discussions on existing problems and mechanisms of available drugs were seldom carried out so far. Therefore, this review would summarize the process of pathological variations in the GABA system under cerebral I/R injury and will cover corresponding probable issues and mechanisms in using GABA-related drugs to illuminate the concern about clinical illness for accurately preventing cerebral I/R injury. In addition, the study will summarize the increasing GABA signals that can prevent I/R injuries occurring in peripheral organs, and the roles of GABA were also discussed correspondingly.

## 1. Introduction

Ischemia-reperfusion is a pathological process from an initial cutoff of blood supply to an organ to the subsequent blood perfusion and the accompanying reoxygenation [1]. Frequently, I/R injury occurs in various clinical settings (e.g., thrombolytic therapy, coronary angioplasty, organ transplantation, aortic cross-clamping, or cardiopulmonary bypass) [2]. In the process, I/R is followed by some biological variations in a wide range of conditions, including vascular leakage, cell death programs (apoptosis, necrosis, and autophagy-associated cell death), autoimmunity (autoantibody and complement activation), innate and adaptive immune activation, no reflow phenomenon, and transcriptional reprogramming [3]. These pathological variations cause I/R injuries of different tissues from one organ injury (e.g., acute coronary syndrome in the heart, acute kidney injury in the kidney, and intestinal I/R injury in the intestine) to multiorgan injuries (e.g., kidney and intestine injuries in trauma and resuscitation, kidney and brain injuries in circulatory arrest, and multiorgan injuries in major surgery). Multiple mechanisms are involved in the process, including free radicals, inflammation [4], the energy metabolism disorder of brain tissues, the toxicity of enhanced excitatory amino acid function, intracellular ion (Ca^2+^) overload [5], the nitric oxide (NO) cytotoxic effect [6], and increased opening of the blood-brain barrier [7]. Three therapeutic strategies for I/R injuries have been proposed, including prior to ischemia (preconditioning), during ischemia (perconditioning), and after ischemia (postconditioning) [8]. Recently, remote ischemic preconditioning has received more attentions as an optimized strategy of protecting against I/R injury in some organs with many advantages of high effectiveness, safety, noninvasiveness, easy application, and cost-effectiveness [9]. In addition, many therapeutic substances for I/R injury have been developed, including endogenous substance supplements (e.g., endogenous gases: NO, carbon monoxide, and hydrogen sulfide [10]), chemical drugs (e.g., natural compounds: cannabinoids [11]; synthetic drugs: cyclosporine [12]), and stem cell therapy [13]. Due to some side effects of applying chemical drugs [14, 15] and high cost of stem cell therapy [16], targeting endogenous molecules or signals may be a better choice. For instance, GABA, an abundant substance in the nervous system, is believed to be related to I/R injury.

GABA, an important neurotransmitter, had actually been synthesized in 1883 before it was found as a metabolite product in the plant and microbe [17]. Until 1950, it was found existent in mammalian brains. Hereafter, GABA was established as its neurotransmitter identity in crayfish [18]. It is now known that GABA primarily plays inhibitory effects on neuronal excitabilities of postsynaptic membrane in the central nervous system of adults [19]. GABA can also relax muscle at neuromuscular synapses [20]. However, in some cases, GABA exhibits excitatory effects (e.g., promoting contraction of enteric muscles in defecation [21] and immature CNS neurons [22]). Therefore, GABA is considered as a necessity to neural developments (e.g., neural progenitor cells and neural stem cells) and normal nervous functions. The disrupted variation of GABA levels in the nervous system may be related to numbers of nervous diseases (e.g., epilepsy [23], Parkinson's disease [24], Alzheimer's disease [25], Huntington's disease [26], psychiatric disorders [27], and stroke [28]) and other disruptions (e.g., cognitive impairment [29] and I/R injury). Besides, due to its existence in peripheral tissues and organs (pancreas, thyroid, gastrointestinal tract, lung, testis, liver, etc.) [30], GABA has also exhibited much broader physiological and pathological roles beyond the nervous system (e.g., protecting pancreas *β*-cell, lowering blood glucose [31], reducing inflammation [32], and alleviating I/R injury [33]). Significantly, I/R injury is a systemic pathological process which can even be involved in some nervous diseases (e.g., stroke [34]) and many peripheral organs (e.g., liver [35]). Consequently, the roles of GABA in I/R injuries of the central nervous system and peripheral organs may be a crossing topic which is receiving more and more attentions.

Over these years, with extensive researches about the physiological role of GABA and potential approaches for preventing I/R injury, the relationship between the GABA system and I/R injury is gradually revealed and some has been partly elucidated. Therefore, it is urgent to discuss emerging issues in the present data to propose topics for further investigations. However, seldom systemic reviews had been published. In this review, recent research progresses about the physiology of the GABA system and the roles of GABA in I/R injuries of the nervous system (mainly in the brain) and peripheral organs as well as the mechanisms are summarized.

## 2. A Brief of GABA Physiology

GABA is primarily synthesized from glutamate by glutamate decarboxylase (GAD) in the body. Two GAD isoforms have been identified: GAD65 and GAD67, which derive from two cDNAs of a single separate gene [36]. Apart from existing in the nervous system, GAD is also distributed in other tissues outside the nervous system including the liver, kidney, pancreas, testis, ova, oviduct, adrenal, sympathetic ganglia, gastrointestinal tract, and circulating erythrocytes [37]. Other sources of GABA are spermine, spermidine, ornithine and putrescine which are deaminated and decarboxylated to produce GABA. GABA packed in the synaptic vesicles by vesicular GABA transporters (VGAT) is released into the synaptic cleft under triggering of Ca^2+^ influx to combine GABA receptors before playing physiological roles [38]. In normal physiology, GABA has difficulties to cross the blood-brain barrier [39], despite some transporters (GABA transporters (GAT)/betaine-GABA transporter) being able of transporting GABA [40].

GABA has three types of receptors, including two ionotropic receptors (GABA_A_ and GABA_C_ receptors) and a metabotropic receptor (GABA_B_ receptor). GABA_A_ and GABA_C_ receptors have various pentamer combinations derived from *α*1-6, *β*1-3, *γ*1-3, *δ*, *ε*, *π*, and *θ* subunits summarized in a review [41]. These subunits play different roles. The two ionotropic receptors mainly activate ligand-gated chloride (Cl^−^) channels to produce hyperpolarization. Potassium (K^+^) chloride cotransporter (KCC2) pulls Cl^−^ out of neurons [42] to keep balance of Cl^−^ contents between inside and outside the neurons. Moreover, GABA_A_ receptor is also the target of many substances (e.g., benzodiazepines, barbiturates, neuroactive steroids, intravenous and inhalational anesthetics, and ethanol [43]), in which benzodiazepines are well known for their applications as positive allosteric modulators of GABA_A_ receptor [44]. GABA_B_ receptor has two subunits (B1 and B2) and is a G protein-coupled receptor, mediating inhibitory effects by activating voltage-gated Ca^2+^ channels, K^+^ channels, and adenylyl cyclase to induce cell hyperpolarization and inhibit neurotransmitter releases [45].

After producing biological effects, most GABA is reabsorbed from synaptic cleft and reused by GAT. GAT has four types: GAT1, GAT2, GAT3, and betaine-GABA transporter [46]. GAT1 and GAT3 are the major types in the brain, regulating the balance of GABA [47]. The GAT1 is especially important in neurons and can also inversely transport GABA out of neurons with uncertain mechanisms [48]. GAT3 is mostly distributed in glial cells [49]. GABA entry into cells is metabolized by two catabolic enzymes: GABA transaminase and succinic semialdehyde dehydrogenase (SSADH). The final metabolic product of GABA, succinic acid, enters into the tricarboxylic acid cycle for energetic supplements [50].

## 3. The Role of GABA in Cerebral I/R Injury in Adult

### 3.1. Variations of GABA Levels, Synthesis, and Metabolism during Cerebral I/R Injury

It is well known that cerebral ischemia injury can cause high mortality and disability [51]. Although the reperfusion is used to restore brain blood supplement, it can also induce further injuries involving a complex pathological progress (I/R injury) [52]. Many mechanisms have been proposed to explain the progress, including toxicity of high glutamate activity, Ca^2+^ overload, oxidative stress, inflammation and apoptosis, inflammation, breakdown of the blood-brain barrier, cerebral infarction, and edema [52–54]. The imbalance including the decreased transmission of GABA signal and the increased excretion of excitatory amino acid (glutamic acid) in I/R [55] indicated that GABA may play special roles in the process of I/R injury. Enhancing GABA actions by different enhancers of GABA transmission can partly restore the balance of GABA and glutamate transmissions, pointing out a potential preventive strategy for cerebral I/R injury. Even an enhanced GABA signal pathway can inhibit glutamate release induced by ischemia, such as treatment with GABA receptor agonists [56], promoting normalization of the two neurotransmitter pathways.

First, accumulating data had shown that GABA levels and the function status of its metabolism are affected differently in different periods of cerebral I/R injury, reflecting that the GABA system can be an important index of cerebral I/R injury and also monitor the restoration of cerebral I/R injury. The most prominent feature in these phenomena is the rise of extracellular GABA levels in most cerebral I/R injuries, while in transient cerebral ischemia, GABA levels return to the normal at the start of reperfusion, or in repeated ischemia, it decreases to the undetected level [57]. Recent studies showed that in animals [58, 59] and humans [60] suffering ischemia attacks, GABA contents in the cerebral hemisphere actually declined. Although the efflux transport of GABA out of the brain via the blood-brain barrier increased in I/R injury [61], reduced GABA contents may mainly result from the variations of GABA synthesis, release, and metabolism. In the early stage of ischemia, GABA release and the inhibition of its metabolism may mainly be responsible for increased GABA levels. Allen et al. found that GABA releasing was induced sequentially by the exocytosis due to the entering of extracellular Ca^2+^ into neurons during the anoxic depolarization, and then, reversed uptake of GABA by GAT1 occurred after the anoxic depolarization [48]. However, upregulated GAT1 expressions under ischemic conditions after a long time started the removal of GABA from the synaptic cleft [62], reducing GABA metabolism. In the aspect of GABA synthesis, the variation of GAD after cerebral I/R has a striking similarity. Moreover, increased expressions of GAD in a short time after I/R (e.g., large aspiny neurons [63]) can produce more GABA and provide the basis for GABA release and reversed uptake of GABA by GAT1 in neurons to increase the survival. After that, the loss of GABAergic neurons [64] and the decrease of GAD protein expression [65] occurred (e.g., in substantia nigra of chronic ischemia rats). At this point, although increased protein expression of GAD (GAD67) may compensate the neuron injury induced by hypoxia-ischemia, elevated GAD expression is also associated with cell death [66], and the compensation is even weaker in the immature brain compared with the mature brain. Another study showed that truncated cleavage product (tVGAD) from VGAD by calpain following focal ischemia had the lower ability in the GABA synthesis-packaging coupled with VGAT, leading to reduced GABA transmission despite its increased activity compared with that of VGAD in the focal cerebral ischemic rat brain [67]. Further, the decreased mRNA level of the VGAT [68] after I/R indicated faster eliminations and slower packages of GABA. However, the protein levels of VGAT in substantia nigra pars reticulate [65] and other brain regions [69] were stable after ischemia by using immunohistochemistry. It may be due to the rapid formation of a stable truncated cleavage product (tVGAT) from VGAT under ischemia by calpain which cannot be discriminated by immunohistochemistry and may further decrease GABA release after a long time [70]. The reason may be that tVGAT is redistributed out of synaptosomes with an unclear mechanism, probably explaining reduced GABA release with more production and redistribution of tVGAD in ischemia [67]. In addition, the protein levels of SSADH and SSAR were reduced in some regions after ischemia [71], indicating that GABA metabolism was disturbed, and due to the downregulated SSADH level, the less supplement of GABA shunt to produce adenosine triphosphate (ATP) in pyruvate dehydrogenase into the tricarboxylic acid cycle aggravated the neuron damages.

### 3.2. Expressions, Binding Abilities, and Signal Transmissions of GABA Receptors during Cerebral I/R

Apart from the normal GABA level in the brain necessary for maintaining neural functions in the cerebral I/R process, GABA receptors and GABA signal transmissions are also very vital. An interesting study had well proved the view of point that only blocking GABA synthesis is not enough to alter neuron functions. A study [72] showed that reduced GABA production by a GAD inhibitor did not alter dendrite growth *in vitro*. So, GABA receptors and their transmissions may be involved in neural I/R injury. Thus far, data about variations of GABA receptors showed the characteristic of time dependence resulting from I/R injury of the mature neuron. A previous study had demonstrated that the level of GABA_A_ receptor in the hippocampus and cerebral cortex was downregulated within 30 min after I/R [73]. In mechanism, the reduced density of GABA_A_ receptor probably accounted for this event. It can result from the internalization of GABA_A_ receptor mediated by dephosphorylation of its *β*3 subunit (serine 408/409) [74], which occurred under increased recycling of GABA_A_ receptor by huntingtin-associated protein 1 [75] and decreased GABA_A_ receptor clustering by downregulating a protein (gephyrin) responsible for the transport and synaptic anchoring of GABA_A_ receptor after oxygen-glucose deprivation [76]. Differently reduced GABA_A_ receptor density was also demonstrated in the optic lobe of an embryonic chicken [77]. Meanwhile, the mRNA levels of GABA_A_ receptor subunits (*α*1 and *α*2) in the hippocampus and dentate gyrus were also downregulated in another study [78]. Besides, at 48 h after transient global ischemia, there were different variations of GABA_A_ receptor subunit (*α*1, *β*2, and *γ*2) mRNA in cerebral cortexes (no change) and caudate putamens (upregulated both subunits) in the rat ischemia model [79], indicating different injuries in different brain regions in the same time. However, despite the confirmed upregulated mRNA levels of GABA_A_ receptor *α*1 and *α*2 subunits [80], protein contents of the *α*1 subunit in rats with brain ischemia induced by the manmade thrombus decreased at 7 days after I/R [81]. In other cases, subunits (e.g., *α*1) of GABA_A_ receptors in the perilesional cortex were highly expressed, which may induce functional reorganization (increase in dendritic-like structures [82]) or other dysfunctions of the nervous system (e.g., epileptogenesis [83]) in cerebral I/R injury. In addition, although the mRNAs of GABA_B_ receptor subunits (B1 and B2) were detected coexisting with parvalbumin in gerbil hippocampus neurons at 4 days after transient global ischemia [84], these neurons surviving the ischemia insult were just a few. *In vitro*, a brief episode of oxygen and glucose deprivation can decrease GABA_B_ receptor contents in the cell surface through activating endoplasmic reticulum stress [85]. Therefore, the fluctuating process of GABA receptor expressions hinted the decline of neuron functions or even death in cerebral I/R injury. In contrast, GABA receptors in young individuals were more vulnerable and caused functional compliments. For instance, the mRNA levels of GABA_A_ receptor *α*1, *β*2, and *γ*2 subunits significantly decreased in the cerebral cortex and caudate putamen in young ischemia rats [79]. These variations in neonatal individuals can directly induce seizure occurrence [86].

On the other hand, the functional status of GABA receptors is also responsible for normal GABA signal transmission. It had been demonstrated that the binding abilities of GABA receptors with ligands declined after ischemia and the binding activity is often characterized by the binding value of special agonists with them. For instance, muscimol, a GABA receptor agonist, had a lower binding value in the ischemic cortical penumbra after transient focal cerebral ischemia I/R [87]. Even after transient oligemia, a significant reduction of GABA_A_ receptor binding values can last for a long time [88]. Conversely, GABA_A_ receptor in surviving neurons of the gerbil hippocampus against I/R had upregulated binding abilities with muscimol according to a research [89]. However, the tolerance of neurons was just limited in a certain period (within 48 h after cerebral I/R).

Moreover, the intracellular signal transmission of GABA receptors also disrupts. Inhibitory postsynaptic potentials induced by GABA had disappeared *ex vivo* [90] or weakened *in vitro* [91] in the short term after ischemia. GABA signal transmission also declined with reduced chloride ion influx *in vivo* [91], *in vitro* [92], and *ex vivo* [90]. Downregulation of KCC2 may be responsible for the results [93], which are unable of restoring Cl^−^ gradient on both sides of the neuron membrane. In a research about long-term injuries after ischemia, the dysfunction of GABA signal transmission was remarkable, despite the normal presynaptic neurons which had survived the I/R injury releasing a normal amount of GABA [94]. This result indicated that GABA transmission after GABA receptors was more fragile. On the other hand, high-resistant neurons still exhibited the normal response ability of the GABA receptor. Zhan et al. found that miniature inhibitory postsynaptic currents and GABA-evoked currents in high-resistant CA1 interneurons after transient forebrain ischemia did not decrease [95], which might delay the I/R injury. Liang et al. further proved that the enhancement of GABA synaptic transmission in CA1 neurons following forebrain ischemia was attributed to the postsynaptic signal dysfunction rather than disrupted GABA releasing in the presynaptic membrane [96]. The research had proved that adenosine A1 receptor activation was a potential reason for the phenomenon. Other factors after ischemia had been proposed accounting for the dysfunction of postsynaptic GABA transmission in a comprehensive review [97], including increase in intracellular chloride ion, accumulation of some signal substances (e.g., Ca^2+^ and eicosanoids), production of reactive oxygen species, and reduction of ATP. In addition, the degradation of tumor suppressor phosphatase and tensin homolog after ischemia had double effects according to a recent research [98]. The molecule can enhance GABA-evoked current to protect neural functions by increasing the expression of GABA_A_ receptor *γ*2 subunit on the one hand and has the converse action of damaging astrocyte functions on the other hand.

### 3.3. The Protective Effects of GABA and Its Enhancers on Cerebral I/R Injury

In view of the importance of GABA in cerebral I/R injury and the difficulty of GABA crossing the blood-brain barrier, some GABAergic drugs had been used for attenuating I/R injury in clinical and animal tests. Early in 2001, Schwartz-Bloom and Sah had reviewed some GABAergic drugs used in I/R animals and few human subjects [97], including GABA agonists (e.g., muscimol, thip, and baclofen), GABA modulators (e.g., benzodiazepines), GABA uptake inhibitors (e.g., tiagabine), and GABA metabolism inhibitors (*γ*-vinyl GABA). However, due to the narrow therapeutic window, only short-term protective effects (about 1 month) against cerebral I/R injury were demonstrated in most researches. Moreover, adverse effects of some drugs aggravating neuronal deaths were frustrating. The discussion of further mechanisms was still limited. Most importantly, selective applications of these drugs and clinical investigations were not discussed due to the shortage of data. In recent years, much broader and deeper studies were illuminating to develop preventive and therapeutic strategies for cerebral I/R through targeting the GABA signal pathway. In this section, an updated review from several aspects of new progresses was made.

#### 3.3.1. The Clinical Effects of GABA on Cerebral I/R Injury

For a long time, barbiturates had clinically been used to control the intracranial hypertension in transient ischemia events in the operating room; some potential side effects (having a depressant effect on the cardiovascular and respiratory systems) might affect the applications (e.g., needing intensive care) [99]. Meanwhile, another system review [100] summarized that GABA receptor agonists (e.g., chlormethiazole) had better effects on patients with acute ischemic (within 12 hours) or hemorrhagic stroke. Some adverse events (e.g., somnolence and rhinitis) also occurred. Recently, the long-term intrathecal treatment of baclofen can improve the functional independence in the traumatic brain injury and stroke groups [101]. However, adjustments of doses were complex and needed further investigations. In addition, GABA enhancer treatments, such as rac-hopantenic acid (pantogam activ) [102, 103] and adaptol [104], remarkably improved psychopathological, psychometric, and detailed somatic functions (e.g., cognitive functions, but also emotional state) with reduced adverse events (e.g., drowsiness and headaches) in patients with chronic cerebral ischemia. Therefore, developing new drugs of GABA enhancers with less adverse effects may be the next focus of clinical studies.

#### 3.3.2. The Relationship between Doses and Effects May Be an Important Reference Point

Effective doses are needed in using GABA enhancers for cerebral I/R injury. For instance, some enhancers of GABA actions (e.g., topiramate and *γ*-vinyl GABA) cannot prevent focal I/R injury induced by the occluding right middle cerebral artery in functional or histological measures at high concentrations of the two drugs (100 mg/kg and 1000 mg/kg, respectively) whether in preconditioning or following ischemia [105]. A later study showed that *γ*-vinyl GABA (30-300 *μ*mol/L) exhibited “U-shaped” effects of antagonizing LDH and glutamate releases induced by hypoxia-glucose deprivation/reoxygenation *in vitro* [106]. In addition, cotreatment may produce better effect despite lower doses. Cotreatments of *γ*-vinyl GABA (30 mg/kg) with purinergic (P2X) receptor antagonists intraperitoneally (i.p.) can prevent neuronal cell death of the gerbil hippocampus at 24 h after I/R [107], and it alone has no effects. Diazepam has also “U-shaped” effects both *in vivo* (0.5-10 *μ*mol/L) [108] and *in vitro* (0.5-5 *μ*mol/L) [109], though its effect was involved in hypothermic neuroprotection [110]. Whether the mechanism is related to the status of the GABA signal or through other pathways may be the next focus.

#### 3.3.3. More Mechanisms Based on or beyond GABA Signal Transmission

With the broader researches of using GABAergic drugs preventing cerebral I/R injury, some strategies are emerging and may produce better effects (e.g., coadministrations of GABA_A_ and GABA_B_ receptors having better effects than given alone in the *in vitro* ischemia model [111]) and less adverse effects (e.g., special activations of GABA subunits: *β*, *α*1, and *γ*; coadministrations of etomidate and propofol; and coadministrations of zolpidem and diazepam [112]).

Moreover, multiple mechanisms had been presented in exploring the effects of different GABAergic enhancers in cerebral I/R (Table 1), including improved expressions of GABA receptors (or subunits); increased NO synthase and postsynaptic density 95 (PSD95) interaction; activated *α*2-*δ* subunit of the voltage-dependent Ca^2+^ channel and Ca^2+^/calmodulin-dependent protein kinase II; upregulated B-cell lymphoma-2 (Bcl-2) expressions; decreased Bcl-2-like protein 4 (Bax)/Bcl-2 ratio; downregulated expressions of procaspase-3 and caspase-3 through glutamate receptor 6 (GluR6)/PSD95/mixed lineage kinase 3 (NLK3)/c-Jun N-terminal kinase 3 (JNK3) and protein kinase M zeta (PKM*ζ*)/KCC2; reduced glutamate and lactate dehydrogenase (LDH) levels; inhibited N-methyl-d-aspartic acid (NMDA) receptor/Src-mediated signal; suppressed autophagy by activating protein kinase B (Akt), glycogen synthase kinase-3 beta (GSK-3*β*), and extracellular regulated protein kinases (ERK) and neutrophil-directed migration in a phosphoinositide 3-kinase- (PI3K-) dependent manner; attenuated oxidative stress; decreased intracellular Ca^2+^ contents; and restored contents of brain-derived neurotrophic factor (BDNF), vascular endothelial growth factor (VEGF), tyrosine kinase B (TrkB), neural cell adhesion molecule (NCAM), and G protein-activated inwardly rectifying potassium (GIRK, Kir3). Among them, most played the roles which may be related to improved GABA transmission. However, individual drugs played the protective effect through some pathways which were irrelated to GABA signals. For instance, diazepam acted on the peripheral benzodiazepine receptor in the outer mitochondrial membrane [108]. Therefore, more potential mechanisms related to GABA signal or other effects in or outside the ischemia regions needed further investigations, and systemic classifications of different GABA enhancers are necessary in the long run.

### 3.4. The Protective Effect of Preconditioned and Delayed Treatments with GABA Transmission Inhibitors on Adult Cerebral I/R Injury

However, in some circumstances, preconditioned and delayed treatments with GABA transmission inhibitors can produce protective effects on adult cerebral I/R injury according to limited studies. In fact, an early study had already some kind of hint. A benzodiazepine receptor partial agonist (PNU-101017) intriguingly exhibited the better effect of preventing hippocampal neuronal degeneration in I/R injury than a full benzodiazepine receptor agonist [129] in preconditioning. The difference was more obvious when the two drugs were given after ischemia. The result was probably due to GABA receptor overactivations after I/R, triggering the outward of bicarbonate over the inward of Cl^−^ and a further depolarization. Recently, some reports further confirmed the effect. First, the treatment with GABA_A_ receptor antagonist (bicuculline) at 1-2 days before oxygen-glucose deprivation made the cultured cortical neurons more tolerant to the insult [130]. The mechanism was related to calmodulin-dependent phosphorylations of ERK1/2 and cAMP-response element binding protein (CREB). Interestingly, alike results were also obtained *in vivo*. In a study, in contrast to attenuating I/R injury by activating GABA transmissions in adults, the treatment with a GABA_A_ receptor *α*5 subunit inverse agonist (L-655,708) at a low dose of 1.5 mg by subcutaneous implantation at 1 week after the focal ischemia induced by cortical microinjection of endothelin-1 can significantly restore motor skills of ischemia stroke rats by inhibiting GABA signal [131]. Recently, L-655,708 treatment (1, 5 mg/kg) only after 3 days after middle cerebral artery occlusion can trigger neurogenesis [132]. It hinted that the targeted inhibition of special GABA receptor subunits may be the key. In another research, Alia et al. found that hampering GABA_A_ transmission after photothrombotic ischemia by a benzodiazepine inverse agonist (DMCM: methyl-6,7-dimethoxy-4-ethyl-beta-carboline-3-carboxylate, i.p., 1.5 mg/kg) can significantly improve motor function [133]. The effect of DMCM may remodel GABAergic inhibitory networks controlling motor function. Although limited reports were presented, more mechanisms about status of GABA receptors and its signals during the period of I/R injury may be responsible for the results. Preconditioned and delayed treatments may also be the key of the effect. Therefore, more accurate effects concerning GABAergic inhibitory drugs need further investigations.

## 4. The Role of GABA in Cerebral I/R Injury in Neonate

As stated above, GABA exhibits excitatory effects in the immature nervous system compared with inhibitory effects in the mature nervous system. Therefore, its effect on cerebral I/R injury of neonate was specially discussed in this section. Thus far, limited studies showed that GABA transmission inhibitors played the protective effects on neonatal cerebral I/R injury. The effects may be due to increased GABA levels and the losses of GABA receptors (subunits) or its pathway expressions [86, 134, 135] occurring in immature I/R injury, hinting possible overactions of the GABA transmission pathways. This point can be demonstrated in a study that a GABA_A_ receptor antagonist (bicuculline) can further enlarge the intracellular Ca^2+^ accumulation under reduction in Cl^−^ gradient across the membrane induced by oxygen-glucose deprivation in neonatal rat neocortex [136]. Therefore, activating GABA_A_ receptor may aggregate the I/R injury in the neocortex of neonatal rats. Moreover, treatments with two GABA_B_ receptor antagonists (CGP 35348 and CGP 55845) can remarkably improve the motor function and inflammatory response of 10-day-old rats subjected to the hypoxia-ischemia stimulation [137–139]. In another study [140], a GABA receptor agonist (topiramate) generated more damage to the brain and the cognitive ability injury in the hypoxic-ischemic brain of neonatal rats (over 7-day age), except for protective effect on hypoxic-ischemic brain injury in rats (1, 4-day age). However, the mechanism of the latter effect was not further explored.

## 5. The Role of GABA in Spinal Cord I/R Injury

Spine cord ischemia is a rare event and difficult to diagnose for its varying degrees of injury. Its clinical manifestations include anterior spinal artery syndrome, posterior spinal artery syndrome, and spinal transient ischemic attacks (e.g., transverse medullary infarction, central spinal infarct, infarction at the level of conus medullaris, and sulcocommissural syndrome) [141]. GABA within the spinal cord should have equal significance as in the brain. However, seldom studies about the role of GABA in spinal cord I/R injury were presented. Limited studies showed that the GABA level significantly decreased [142, 143] and spontaneous inhibitory postsynaptic currents induced by GABA increased [144] after spinal cord I/R, hinting the overactivation of GABA signal transmission. However, inconsistent results according to the present data showed that both GABA agonists and antagonists can restore spinal cord I/R injury. For instance, the former (intraperitoneal treatment with vigabatrin [145] and intrathecal treatments with baclofen and nipecotic acid [146]) can restore histopathologic injury, spasticity, and rigidity in rats after transient spinal cord ischemia. The latter (i.v. bicuculline and semicarbazide [147]) mainly restored reflex potentials after spinal cord ischemia in cats. Thus, the complex mechanism of the role in spinal cord I/R injury exists and needs further distinguishing.

## 6. Roles of GABA in I/R Injuries of Other Organs outside of the Nervous System

### 6.1. GABA and Gastric I/R Injury

Gastric ischemia-reperfusion is a common clinical problem associated with various physiopathological stress conditions such as surgery, hemorrhagic shock, peptic ulcer bleeding, vascular rupture, and ischemia gastrointestinal disease, which may cause the damage of the gastric mucosa [148]. In these processes, the dysfunction occurring in the neuroendocrine system can induce increased catecholamine levels, vasoconstriction, decreased cardiac output, reduced blood volume, and the release of proinflammatory factors [149], which promote flow of blood into the heart, brain, and muscles from the gastrointestinal tract and skin [150]. So, the central nervous system may play a vital role in gastric I/R injury. Nowadays, fastigial nucleus (FN), hypothalamic paraventricular nucleus (PVN) [151, 152], and lateral hypothalamic area (LHA) [153] are thought as major regions regulating gastric activity and gastric mucosal injury in gastric I/R injury. The GABA system, abundant in the central nervous system, was proved playing a vital role in attenuating gastric I/R injury. Zhu et al. [154] found that microinjecting glutamate into the interpositus nucleus (IN) of the cerebel can attenuate gastric I/R injury, decrease discharge frequency and intensity of greater splanchnic nerve (GSN), and improve blood flow, apoptosis, and proliferation of rat gastric mucosa. The effect can be reversed by the pretreatments of microinjecting 3-mercaptopropionic acid (GAD inhibitor) into IN or bicuculline into LHA. The result suggested that GABAergic neuron in IN acted on its receptors in the lateral hypothalamic area and subsequently inhibited the activity of the GSN innervating stomach. Its dysfunction was considered as an important promoter of gastric I/R injury progression. Also, GABAergic neurons in FN of the cerebellar also participated in attenuating gastric I/R injury through the cerebellar-hypothalamic circuit [155]. Another study [156] further proved that high levels of GABA_A_ receptor in the LHA of rats could be responsible for significantly alleviated gastric I/R injury by the overexpression of the receptor induced by adenoviral vector. Reduced norepinephrine and angiotensin II levels in plasma and peripheral GSN activity marked multiple ways of GABA preventing against gastric I/R injury in the study. A recent investigation [157] showed that the GABA_B_ receptor in the LHA was involved in the protective effects on the gastric I/R injury caused by clamping the celiac artery. The administration of GABA_B_ receptor agonist (baclofen) can significantly attenuate gastric I/R injury. Interestingly, significantly reduced oxidative stress indexes (e.g., nicotinamide adenine dinucleotide phosphate-oxidase (NOX2, NOX4)) and attenuated inflammation (IL-1*β*) in the gastric mucosa by baclofen treatment were observed in model rats. Although the pathway of baclofen attenuating gastric I/R injury was probably through decreasing expressions of NOX2/NOX4, the neuroendocrine effect mediated by neurotransmitter calcitonin gene-related peptide also played the important role because the neurotransmitter is released by neurons innervating the stomach from the dorsal root ganglion under the injection of baclofen into the LHA. Therefore, GABA or its agonists may have the prospect of curing gastric I/R injury. However, the direct effect of GABA in gastric I/R is still unclear.

### 6.2. GABA and Intestinal I/R Injury

Intestinal ischemia-reperfusion (IIR) injury occurs in numerous clinical events following intestinal ischemia, such as superior mesenteric artery occlusion, hemorrhagic shock, intestinal and liver transplantation [158, 159], neonatal necrotizing enterocolitis [160], volvulus [161], trauma [162], and cardiopulmonary disease [163]. IIR injury can induce the damage and dysfunction of multiple organs [164]. Although improved treatments were recently applied to clinical tests, the morbidity and mortality still remained high [165]. Studies about the effect of GABA on IIR injury had just begun. A recent study [166] showed that IIR resulted in a significant decrease of intestinal innate immunity indexes (immunoglobulin A, alpha-defensin-5, and antioxidative enzyme) in Peyer's patch cells of the rat small intestine, and GABA pretreatments (oral administration, 30 mg/kg) significantly produced the protective effects on IIR injury in which mRNA levels of R alpha-defensin-5 and superoxide dismutase 1, 3 together with immunoglobulin A levels were increased *in vitro*. In addition, GABA receptor activations in rat focal cerebral I/R injury seem to affect intestinal flora [167], suggesting that indirect effect of GABA on IIR may exist. Therefore, GABA's indirect effect and clinical data about applying GABA or its agonists to prevent or cure IIR injury should be focused in the future.

### 6.3. GABA and Renal I/R Injury

I/R injury is primarily responsible for acute kidney injury, which can cause high mortality and morbidity and elevated cost of treatment [168, 169]. Renal I/R injury is the restoration of blood reperfusion after the blocking of renal blood flow, which often occurs in surgeries of vascular and cardiac, trauma, and kidney transplantation [170, 171]. GABA may play important roles in renal physiology and pathology [172]. For instance, GABA has been reported to regulate blood pressure by increasing excretion of water and sodium ion (Na^+^) in the kidney *ex vitro* [173]. Recent studies showed that GABA can attenuate renal I/R injury from two pathways of the central nervous system and the peripheral nervous system. Kobuchi et al. found that intravenous injection of GABA (10 and 50 mmol/kg) can dose-dependently suppress the elevated activity of renal sympathetic neuron and the venous norepinephrine overflow in the rat renal I/R model [33]. Selective treatment using GABA_B_ receptor antagonist (CGP52432) can reverse the protective effect of GABA on renal I/R injury, hinting that GABA_B_ receptor is the targets of GABA action. Then, GABA_A_ receptor antagonist picrotoxin had been found aggravating the acute kidney injury induced by I/R in investigating the mechanism of sodium valproate (reducing GABA degradation) protecting against renal I/R injury [174], suggesting that GABA_A_ receptor directly plays the protective effect of GABA on renal I/R injury compared with the little, no, or opposite effect of GABA_B_ receptor in the condition of GABA shortage. But a recent study [175] showed that GABA_A_ receptor antagonist (bicuculline) treatment by the intracerebroventricular administration was unable to prevent the preventive effect of GABA on renal I/R injury. Conversely, renal I/R injury could be prevented by GABA_B_ receptor agonist (baclofen), indicating that activated GABA_B_ receptor in the central nervous system was responsible for the protective effect. In addition, the effect of GABA on renal I/R injury exhibited gender-related difference. GABA treatment can attenuate pathological renal parameters (blood creatinine and urea nitrogen, kidney weight, and renal level of malondialdehyde) in female rats rather than male rats [176]. However, it was intriguing that GABA was unable to protect renal I/R injury in ovariectomized rats whether estradiol was administrated or not [177]. The result suggested that variations of the internal environment in different physiological states may interrupt the effect of GABA on renal I/R injury. Therefore, more complex internal environments need considerations.

### 6.4. GABA and Hepatic I/R Injury

Hepatic I/R injury is a systemic pathological process which induces not only hepatic function and irreversible injury but also cascade of dysfunctions of other organs [178]. Hepatic I/R injury occurs in some clinical scenarios, including liver resection (e.g., the Pringle maneuver and the hepatic vascular exclusion in liver surgery), liver transplantation, and trauma [179–181]. Two types of hepatic I/R injuries have been proposed. The preservation and storage of the liver before transplantation can cause cold I/R injury [182]. Liver surgery, trauma, setting of transplantation, etc. can induce warm I/R injury due to transient blockage of blood flow [183]. A lot of mechanisms are involved in hepatic I/R injury, including oxidative stress, inflammation, respiratory chain dysfunction of mitochondria, Kupffer cell activation, upregulated vascular cell adhesion molecule, and polymorphonuclear neutrophil injury [184]. Some substances with antioxidative and anti-inflammatory activities had demonstrated protective effects against hepatic I/R injury [178]. Recently, novel targets for attenuating hepatic I/R injury were also proposed (e.g., peroxisome proliferator-activated receptor gamma and several noncoding RNAs) [185]. However, the mechanism remained unclear. Present data showed that GABA was associated with hepatic I/R injury. First, it had shown that GABA may play an important role in monitoring hepatic I/R injury. According to a clinical study about 18 patients, GABA levels in the grafts for liver transplantation immediately after the reperfusion with an isotonic solution for 48 hours were remarkably decreased and then stabilized at baseline values [186]. Thus, GABA can be an index predicting the changing process of a normal functioning liver graft and may participate in protecting hepatic I/R injury. Further studies showed that GABA agonists can attenuate hepatic I/R injury. An animal research showed that pretreatment of donor rats *in vivo* by GABA_A_ receptor agonist (muscimol) before the liver graft surgery significantly reduced the liver cold ischemia/warm reperfusion injury and oxidative stress after the orthotopic liver transplantation [187]. At the end of 6 hours, after the orthotopic liver transplantation with the same treatment on recipient rats according to the donor rats, PI3K, Akt, antioxidant enzymes (superoxide dismutase), ataxia-telangiectasia mutated kinase (ATM), and phosphorylated histone H2AX (gamma H2AX) were also greatly enhanced. The final index as the markers of DNA damage indicated the potential pathway that GABA attenuated hepatic I/R injury. *In vitro*, the treatment to the harvested graft of donor rats with GABA receptor agonists for 4 h of cold ischemia cannot prevent hepatic I/R injury in the orthotopic liver transplantation model or shear stress in the split orthotopic liver transplantation model [188], suggesting that GABA had no direct effect on hepatic I/R injury. In addition, a noteworthy study indicated that flumazenil, an antagonist of benzodiazepine receptors, can improve hepatic encephalopathy produced by hepatic I/R injury [189]. Benzodiazepines are a class of drugs targeting GABA_A_ receptor and enhancing GABA to produce effects which are sedative, hypnotic, anxiolytic, anticonvulsant, etc. [190]. The result that flumazenil attenuates the cerebral complication of hepatic I/R injury may be due to its direct interaction with GABA receptor. A recent research had confirmed that *α*1-*γ*2 interface of GABA_A_ receptor may be the action site of flumazenil [45]. But it was still uncertain whether the effect of flumazenil was related to inhibited GABA pathway mediated by blocking the benzodiazepine signal pathway. In the meanwhile, the effect of flumazenil on hepatic I/R injury had not been explored. Therefore, it was unclear whether it is consistent concerning effects of flumazenil on hepatic I/R injury and its complications (e.g., hepatic encephalopathy). The difference between the probably inhibited GABA pathway and other GABA receptor agonists in attenuating hepatic I/R injury needs further investigations.

### 6.5. GABA and Myocardial I/R Injury

Myocardial ischemia refers to coronary blood flow reduction which cannot meet their metabolic requests. Myocardial ischemia is characterized by loss of oxygen, accumulations of hydrogen ions and lactate, and ion changes (the decline of K^+^ and the increase of Na^+^) in the myocardial extracellular fluid [197]. Myocardial ischemia can lead to arrhythmias, sudden death, myocardial infarction, aneurysms, ruptures, and valvular dysfunction of the heart [198]. Reperfusion is the only effective treatment to prevent the pathological process. However, reperfusion has some deleterious consequences including oxygen radicals, Ca^2+^ loading, and inflammations, which further caused reperfusion-induced arrhythmias, myocardial stunning, microvascular obstruction, and lethal myocardial reperfusion injury [199]. Thus, it is necessary to develop new strategies to prevent the harmful events. GABA has been detected in sympathetic premotor neurons of the medullary raphe controlling sympathetic tone to the heart [200]. Thus, GABA may provide a potential outlet to attenuate myocardial I/R injury through the nervous system. Thirty years ago, Meerson et al. had found that GABA accumulation in the brain by administering the inhibitor of GABA transaminase (sodium valproate) prevented the total duration of arrhythmias and cardiac fibrillation in acute I/R of conscious rats with closed chests [201]. Recently, two studies [202, 203] indicated that the GABA pathway in the nervous system was involved in the cardioprotection against myocardial I/R injury induced by the acute sleep deprivation. Bicuculline, a GABA_A_ receptor antagonist, can abolish the effects, and the mechanisms were related to repressing oxidative stress, NO production, and inflammation. In another hand, how GABA can directly affect myocardial I/R injury should also receive special attentions due to confirmed GABA receptors in the heart [204]. Several other studies (Table 2) showed that different benzodiazepine receptor agonists able of enhancing GABA signals remarkably improved indexes of myocardial I/R injury in several ways, including reducing Ca^2+^ influx, oxidation, ATP-dependent potassium (K(ATP)) channels, and protein kinase C. However, it was likely that these benzodiazepines attenuated myocardial I/R injury not through the GABA signal pathway or at least not directly through acting on cardiac muscle because it was irreproducible in the isolated heart according to a study [191]. Whether other benzodiazepine receptor agonists targeted peripheral GABA receptors in myocardial cells needs further investigations. In addition, direct effects of GABA on myocardial I/R injury had not been explored.

### 6.6. Similarities and Differences about the Potential Effects of GABA on I/R Injury in Different Peripheral Organs

Due to microcirculatory dysfunction by I/R potentially triggering multiple organ injuries [3], it is vital to sum up characteristics about the effects of GABA on I/R injury in different peripheral organs (stomach, intestine, kidney, liver, and heart) before appropriately applying GABA or GABA-related drugs in defending I/R injuries. First, in the similarity, the results about the protective effect of GABA on I/R injuries of peripheral organs from the central nervous pathway were consistent, but the role difference between GABA receptors was not utterly identified, needing further investigations. Further, the direct effects of GABA or GABA-related drugs on different peripheral organs were diverse. For instance, there were no direct protective effects of GABA or GABA-related drugs on I/R injuries of the stomach and liver, compared with direct protective effects on kidney I/R. However, it was particularly noteworthy that flumazenil, an antagonist of benzodiazepine receptors, can aggregate hepatic I/R injury with the utterly unclarified mechanism which differed from the protective effects of GABA enhancers. In addition, the effects of GABA on I/R injuries of the intestine and heart needed to be further certified.

## 7. The Linkage of GABA Molecular Pathway between Central Nervous System and Peripheral Organs in Affecting I/R Injury

In applying GABA-related drugs to attenuate I/R injury, both roles of GABA in the central nervous system and peripheral organs should be carefully considered in order to avoid inconsistent consequences. According to what was mentioned above, some common molecular pathways in the two regions by GABA enhancers had been identified, including increased signal pathways (e.g., PI3K and AKT), reduced signal pathways (e.g., ROS and inflammation), and restored calcium balance. Although there was still shortage of data in peripheral organs, promising prospects with further researches would be possible for simultaneously improving I/R injuries of the central nervous system and peripheral organs by GABA-related drugs. However, more GABA molecular pathways linking the two regions need be certified. Besides, the effects that would happen to these common GABA molecular pathways of peripheral organs after applying GABA transmission inhibitors to attenuate adult cerebral I/R injury should also be paid attention in next investigations.

## 8. The Necessity to Combine GABA-Related Drugs and Molecular Pathways

Due to many GABA-related drugs able to cross the blood-brain barrier resulting in possible dual effects on I/R injuries of the central nervous system and peripheral organs by peripheral administrations (e.g., possible effects of some GABA-related drugs by i.p. on peripheral organs in Table 1), it is essential to combine the drug therapy with molecular pathways and the effects in applying GABA-related drugs to I/R injuries of the two regions. Based on this, it was equally important to further confirm accurate common molecular pathways in protective roles on the two regions and exclude inconsistent effects for other molecular pathways in applying GABA-related drugs on I/R injuries of the two regions in the future.

## 9. Conclusion

I/R insults different tissues and organs so that it is still difficult to protect against I/R injuries according to present data. Developing and perfecting potential protective targets against I/R injury are a more effective strategy. GABA, one of the most abundant neurotransmitters, plays inhibitory effect by targeting its receptors (A, B, and C) to generate hyperpolarization potentials in the adult brain compared with excitatory effects in the immature brain. The increasing number of researches concerning reduced GABA transmission compared with increased glutamate signals occurring in cerebral I/R indicated that GABA could play the key role in the pathology of cerebral I/R injury. Therefore, most researches about GABA and I/R injury focus on the nervous system. In this review, the variations of GABA levels, syntheses, releases, mechanisms, receptors, and signal transmissions occurring during the cerebral I/R process were firstly reviewed from a comprehensive aspect. Alike variations from elevation to reduction in these indexes of the mature brain during I/R injury and the confirmed mechanisms probably provided evidences for the treatment window period. Therefore, strengthening the GABA transmission was a common scheme to prevent cerebral I/R injury in adult. Blocking of the GABA transmission was the method to attenuate cerebral I/R injury of the immature brain. However, new problems are gradually emerging in accumulating studies applying the enhancers of GABA transmission to prevent against cerebral I/R injury, such as dose-effect relationships, beneficial effects of applying antagonists, and multiple mechanisms. First, the relationship between the dose and the effect was a traditional issue and may be resolved by the adjustment of different doses or cotreatments of several lower doses of different drugs. Moreover, some mechanisms in applying different GABA transmission enhancers to attenuate the cerebral I/R injury were found, including improved expressions of GABA receptors; increased NO synthase and PSD95 interaction; activated *α*2-*δ* subunit of the voltage-dependent Ca^2+^ channel and Ca^2+^/calmodulin-dependent protein kinase II, enhanced proliferation (upregulated Bcl-2 expressions and Bcl-2/Bax ratio); inhibited apoptosis (downregulated expressions of procaspase-3 and caspase-3) through different pathways (GluR-PSD95-MLK3-JNK3 and PKM*ζ*/KCC2); reduced glutamate and LDH levels, inhibiting NMDA receptor/Src-mediated signal; suppressed autophagy by activating Akt, GSK-3*β*, and ERK; neutrophil-directed migration by PI3K; attenuated oxidative stress; decreased intracellular Ca^2+^ contents; and restored contents of BDNF, VEGF, TrkB, NCAM, and Kir3. However, more mechanisms and the accurate application of these drugs in different statuses may be the next challenges for investigators. In contrast, pretreatments or delayed treatments with antagonists to block GABA transmission instead restored motor functions *in vivo* and increased the tolerance to oxygen-glucose deprivation *in vitro*. These results are obviously different from the results of previous studies in applying enhancers of GABA transmission. The mechanisms are related to calmodulin-phosphorylation of ERK1/2 and targeting GABA_A_ receptor *α*5 subunit, respectively. These researches indicated that the adjustment to the status of different steps in GABA transmission may immunize the cerebral tissue or later repair neurons. In spinal cord I/R injury, the role of GABA had bidirectional effect, making the application more complex and arousing more attentions in the future.

In addition, the roles of GABA in peripheral organs had also been unearthed, including the stomach, intestine, kidney, liver, and heart. As a whole, enhancing GABA functions by GABA or its agonist can prevent I/R injury despite insufficient data (especially in the clinic) through different pathways. The protective effects of GABA were proved dependent on activated nervous functions, such as the stomach, kidney, and heart. In the intestine and liver, the treatment of GABA agonists can directly attenuate I/R injury. However, the corresponding mechanisms in these organs were seldom reported, whether in test models or in the clinic. Besides, during the I/R injury of multiple organs, the applications of drugs (agonists or antagonists based on GABA transmission) should be considered in the whole body. Consequently, in the view of this point, present data are far from enough for applying GABA to clinic treatments.

## Figures and Tables

**Table 1:** Protective effects of GABA and its agonists on several cerebral I/R injuries and the mechanisms.

Drugs and dose	Treatments	Effects and mechanisms	References
GABA, *γ*-vinyl GABA (30-300 mol/L) *in vitro*	At 90 min after oxygen-glucose deprivation and reperfusion in rabbit brain slices	Reducing the release of glutamate and LDH and tissue water gain (GAT inhibitors)	[106]
Diazepam (10 mg/kg)	Injection (i.p.) at 30 and 90 min after transient gerbil brain ischemia	Reducing excitotoxic and oxidative stress by the peripheral benzodiazepine receptor in the outer mitochondrial membrane (GABA_A_ receptor agonist)	[108]
Zolpidem (0.5, 1.0 mg/kg)	Injection (i.p.) following ischemic stroke in rats	Increasing numbers of cells containing BDNF (GABA_A_ receptor agonist)	[113]
Propofol (20 mg/kg/h, 2 h)	Injection (i.p.) at the onset of reperfusion in the rat model of middle cerebral artery occlusion	Increasing the number of survival neurons and the expression of KCC2, extruding Cl^−^ by upregulating the activity of the PKM*ζ*/KCC2 pathway (GABA_A_ receptor agonist)	[114, 115]
JM-1232 (500 *μ*M) *in vitro*	During rat oxygen-glucose deprivation and reoxygenation	Reducing cell mortality in pyramidal neurons by GABA_A_ receptor/decreased intracellular Ca^2+^ and other mechanisms	[116]
Tramadol (10, 20 mg/kg)	Injection (i.p.) before forebrain I/R in rats	Attenuating postischemic motor impairment by reducing the lipid peroxidation (GABA_A_ receptor agonist)	[117]
Topiramate (80 mg/kg/day, twice daily)	Injection (i.p.) after occlusion of the bilateral carotid arteries in gerbils	Decreasing neurological deficit, attenuating neuronal loss by decreasing the expressions of procaspase-3, caspase-3, Bax/Bcl-2 ratio, GABA_A_ receptor *α*1 and *γ*2 subunits, and KCC2	[118]
Muscimol (1 mg/kg) and baclofen (20 mg/kg) alone	Injection (i.p.) at 6 hours and 1 day after rat I/R	Protecting neurons against death induced by I/R by enhancing NO synthase (GABA_A,B_ receptor agonists)	[119]
Muscimol (1 mg/kg) and baclofen (20 mg/kg) together	Injection (i.p.) at 30 min before I/R in rats	Inhibiting NMDA receptor/Src-mediated signal amplification (GABA_A,B_ receptor agonists)	[120]
Muscimol (1 mg/kg) and baclofen (15 mg/kg) together	Injection (i.p.) to rats at 30 min before ischemia	Neuroprotective effect by PI3K/Akt pathway (GABA_A,B_ receptor agonists)	[121]
Muscimol (1 mg/kg) and baclofen (20 mg/kg) alone or together	Injection (i.p.) before I/R of rats	Attenuating the excitatory JNK3 apoptosis by increased assembly of the GluR6-PSD-95-MLK3 signaling (better combining effect of GABA_A,B_ receptor agonists)	[122]
Baclofen (20 mg/kg) and muscimol (1 mg/kg) together	Injection (i.p.) at 30 min before rat ischemia	Increasing interaction of NO synthase with PSD95 and inhibiting protein phosphatase activity (combining effect of GABA_A,B_ receptor agonists)	[119]
R-Phenibut (10, 50 mg/kg)	Injection (i.p.) at 2 h and 7 days following reperfusion in transient middle cerebral artery occlusion in rats	Improved histological outcome and reduced brain volume; increase of BDNF and VEGF gene expressions (by activating GABA_B_ receptor and *α*2-*δ* subunit of the voltage-dependent Ca^2+^ channel)	[123]
Baclofen (50 mg/kg)	Injection following (i.p.) ischemic insult in the ischemic gerbils	Preventing the loss of hippocampal CA1 pyramidal cells and calmodulin-dependent protein kinase II but not memory deficits (GABA_B_ receptor agonist)	[124]
Baclofen (1.25, 2.5 mg/mL)	Two weeks after chronic cerebral hypoperfusion rats (i.p.)	Increasing Bcl-2/Bax ratio and activating Akt, GSK-3*β*, and ERK which suppressed autophagy (GABA_B_ receptor agonist)	[125]
Baclofen (25 mg/kg)	Injection (i.p.) at 17 d after permanent occlusion of the bilateral common carotid arteries in rats	Improving GABA_B2_ receptor expressions in neural surface, restoring the levels of BDNF, TrkB, and NCAM, and reversing the increased surface expressions of Kir3 (GABA_B_ receptor agonist)	[126]
Baclofen (0.5, 2, and 5 *μ*mol/L) *in vitro*	At 1 hour from the beginning of I/R in rat hippocampal slice cultures	Neuroprotection (GABA_B_ receptor agonist)	[127]
Baclofen (500 mg/L)	Pretreatments (i.p.) before rat I/R	Stimulating neutrophil-directed migration by PI3K (GABA_B_ receptor agonist)	[128]

**Table 2:** Protective effects of GABA receptor enhancers on myocardial I/R injuries and the potential mechanisms.

Species	I/R model	Drugs and dose	Effects and mechanisms	References
Wistar rats: *in vivo* and *in vitro*	Ischemic 20 min, reperfusion 5 min	Phenazepam preconditioning 1 mg/kg	Anticonvulsant and antiarrhythmic effect by central nervous system	[191]
Chick cardiomyocyte *in vitro*	Ischemia 1 h, reoxygenation 3 h	Flumazenil preconditioning 10 *μ*mol/L	Inhibiting ROS/mitochondrial K(ATP) channel	[192]
Chick cardiomyocyte *in vitro*	Ischemia 10 min, reoxygenation 10 min	Flumazenil preconditioning 10 *μ*mol/L	Inhibiting protein kinase C/mitochondrial K(ATP) channel	[193]
Chick embryonic cardiomyocyte *in vitro*	Hypoxia 1 h, reoxygenation 3 h	Diazepam preconditioning (100 *μ*mol/L)	Inhibiting protein kinase C epsilon	[194]
Isolated rat heart *ex vitro*	LPS-induced ischemia 5 h, reoxygenation 20 min	Diazepam in reperfusion (3.0 *μ*g/mL)	Myocardial functional parameters and coronary flow	[195]
Rat cardiomyocytes *in vitro*	Hypoxia 1 h, subsequent reoxygenation	Clonazepam in reoxygenation (100 *μ*mol/L)	Ca^2+^ accumulation by reducing Ca^2+^ influx and preserves mitochondrial membrane potential	[196]

## Abbreviations

Akt: Protein kinase B
ATP: Adenosine triphosphate
Bax: Bcl-2-like protein 4
Bcl-2: B-cell lymphoma-2
BDNF: Brain-derived neurotrophic factor
CREB: cAMP-response element binding protein
ERK1/2: Extracellular regulated protein kinases 1/2
FN: Fastigial nucleus
GABA: *γ*-Aminobutyric acid
GAD: Glutamate decarboxylase
GAT: GABA transporters
GIRK, Kir3: G protein-activated inwardly rectifying potassium
GluR: Glutamate receptor
GSK-3*β*: Glycogen synthase kinase-3 beta
GSN: Greater splanchnic nerve
IIR: Intestinal ischemia-reperfusion
IN: Interpositus nucleus
I/R: Ischemia-reperfusion
JNK: 3c-Jun N-terminal kinase 3
KCC2: Potassium chloride cotransporter
LDH: Glutamate and lactate dehydrogenase
LHA: Lateral hypothalamic area
NCAM: Neural cell adhesion molecule
NLK3: Mixed lineage kinase 3
NMDA: *N*-Methyl-d-aspartic acid
NO: Nitric oxide
PI3K: Phosphoinositide 3-kinase
PKM*ζ*: Protein kinase M zeta
PSD95: Postsynaptic density 95
PVN: Paraventricular nucleus
SSADH: Succinic semialdehyde dehydrogenase
tGAD: Truncated glutamate decarboxylase
TrkB: Tyrosine kinase B
tVGAT: Vesicular GABA transporters
VEGF: Vascular endothelial growth factor
VGAT: Vesicular GABA transporters.
